# Interpersonal discrimination experiences in outpatient care are associated with non-adherence – results of a population survey in Germany

**DOI:** 10.1186/s12889-025-23951-2

**Published:** 2025-08-07

**Authors:** Jens Klein, Anna Christin Makowski, Demet Dingoyan, Olaf von dem Knesebeck

**Affiliations:** Institute of Medical Sociology, University Medical Center Hamburg- Eppendorf, Hamburg, 20246 Germany

**Keywords:** Discrimination, Non-adherence, Non-compliance, Social determinants, Outpatient care, Population survey

## Abstract

**Background:**

Perceived discrimination is known to be associated with adverse health. However, there is a lack of research on detailed experiences of interpersonal discrimination in health care settings and its relationship with health care outcomes in the general population. The objective was to examine the association between perceived interpersonal discrimination experiences in outpatient care and different issues of non-adherence in a population sample in Germany.

**Methods:**

Analyses were based on a cross-sectional online survey conducted in Germany. The sample (*N* = 3,246) was randomly drawn from a panel including the adult population. To assess discrimination, the Discrimination in Medical Settings Scale which originally based on the Everyday Discrimination Scale was used. Five items regrading courtesy, respect, unequal treatment, reserved and dismissive behaviour were introduced to measure the frequency of respective experiences. Patient non-adherence was asked with three questions. Bi- and multivariate analyses adjusted for social characteristics (sex, age, migration history, education, income, health insurance) were conducted.

**Results:**

Results showed that reporting discrimination experiences was significantly associated with all indicators of non-adherence (not following doctor’s advice, not coming for a follow-up visit, not filling a prescription or obtaining recommended drugs). Moreover, there was a trend indicating the more discrimination experiences reported, the higher the likelihood to be non-adherent. Significant odds ratios in the fully adjusted models vary between 1.77 and 2.58.

**Conclusions:**

The present study highlights a critical issue regarding health care delivery in Germany and contradicts the goals of health equity. Discrimination reinforces illness behaviour that is known to be detrimental for treatment outcomes. Tackling interpersonal discrimination and stereotype threat in health care should be a major goal in medical education and training. Physician communication skill trainings have been found to positively influence patient adherence which is an important predictor for health outcomes.

**Supplementary Information:**

The online version contains supplementary material available at 10.1186/s12889-025-23951-2.

## Introduction

Patient adherence was defined by the WHO as “the extent to which a person’s behaviour – taking medication, following a diet, and/or executing lifestyle changes – corresponds with agreed recommendations from a health care provider” [1]. In the past two decades, the term ‘compliance’ was more and more replaced by ‘adherence’ as the latter means a more proactive patient behaviour and underlines an agreement rather than obeying in a more proper doctor-patient relationship [2–4]. Moreover, patient adherence can be considered as a measure of quality of care. Donabedian [5] classified outcomes of health care quality measures which include behaviours related to managing a current illness or influencing future health, such as adherence to health care regimes and changes of health-related practices. Subsequently, the US-American Centers for Medicare & Medicaid Services selected medication adherence as a quality measure to highlight that lack of adherence is a major barrier to attain treatment objectives [6]. Also research suggested the utility of medication adherence as a quality measure [7] based on a clear positive relationship between patient adherence and treatment and health outcomes [7–9]. Various determinants of low patient adherence were found including a low socioeconomic status (SES), female sex, younger age, migration history, and inadequate health care coverage [10, 11]. Within the concept of person-centeredness, the continuity of care as well as a respectful therapeutic alliance between patient and provider are essential elements [12]. Hausmann et al. [13] presumed a theoretical path from perceived discrimination to patient disengagement in terms of following recommendations, returning for future care, and medication adherence. Discrimination is known as differential and disadvantaged treatment of stigmatized individuals or groups [14]. It can occur on an interpersonal (i.e. discrimination between individuals) or structural (i.e. discrimination by policies, regulations, etc.) level. Various studies have shown associations between perceived discrimination and worse mental and physical health outcomes [15–18]. Similar to patient adherence, research on perceived discrimination in health care found associations with lower SES, female sex, younger age, and migration history of the respondents [19–21]. In terms of health care outcomes, associations of discrimination with reduced or inadequate health care utilization and with reduced quality were shown [22–26]. Reported reasons for perceived discrimination were ethnicity, colour of skin, country of origin, sex/gender, age, weight, appearance, SES, specific diseases or disabilities [18–21, 27, 28].

The few findings regarding associations between discrimination in health care and patient adherence among the general population showed significant negative correlations in two U.S. studies [29, 30]. Further existing studies examining these associations were mainly conducted in specific patient populations, came predominantly from the U.S., explicitly surveyed discrimination based on ethnicity, or focused on medication adherence. In view of this lack of research, the present study examined the association between different experiences of discrimination in outpatient care and patient adherence in a sample of the general population in Germany.

## Materials and methods

### Study design and population

Analyses were based on a cross-sectional online survey that was conducted by an established social research institute (forsa) from 18th February to 2nd March 2024. An adult population sample (age ≥ 18 years) was randomly drawn from a panel. This panel comprises a sample of the population living in Germany which was recruited via telephone using a dual-frame approach that included landline and mobile phone numbers. It is regularly refreshed and consisted of about 150,000 people. 8,025 German-speaking individuals were randomly selected from the panel and invited to take part in the present survey via email. After two reminders, data of *N* = 3,246 individuals were collected. The sample was weighted for age, sex, federal state, and education according to the official statistics provided by the Federal Statistical Office of Germany to adequately meet the adult population in Germany according to these sociodemographic characteristics [31]. All procedures were in accordance with the ethical standards of the institutional and/or national research committee and with the 1964 Helsinki Declaration and its later amendments or comparable ethical standards. Initially, participants were asked whether they are generally interested in taking part in the online panel of forsa. During the registration process, a link is provided to the privacy policy and it is pointed out that participation in the panel is voluntary and can be terminated at any time. Inclusion in the panel already constitutes consent to participation, invitation and involvement. For each individual participation in a survey, the respondents explicitly agree to participate in the survey. Thus, participants who agreed gave their consent once again by starting the online survey. The privacy policy can be accessed at any time on every page in the participant area and the surveys of forsa via a button at the bottom of the screen. The survey was approved by the Local Psychological Ethics Committee at the Center for Psychosocial Medicine, University Medical Center Hamburg-Eppendorf (No. LPEK-0719).

### Measures

Patient adherence in outpatient care was surveyed with three questions based on previous research [30]: (1) “In the last 12 months, have you failed to follow a doctor’s advice or a treatment plan?”, (2) “In the last 12 months, have you failed to go to an agreed follow-up appointment with your doctor?”, and (3) “In the last 12 months, have you failed to fill a prescription or obtain recommended medication?”. Answer categories were “yes”, “no”, “don’t know”/”not specified” and “not applicable”. The latter was indicated when respondents did not receive any advice or treatment plan, no follow-up appointment was made, or no medications were prescribed/recommended in the last 12 months. Participants without any need for medical care and without doctor advice/follow-up visit/prescribed or recommended drugs in the past 12 months were excluded from the analyses. Measures of interpersonal discrimination experiences in health care were based on the Everyday Discrimination Scale (EDS) [32]. Originally, the scale consisted of nine items and was later modified for the specific context of discrimination in health care including seven items and satisfactory psychometric properties (Discrimination in Medical Settings Scale (DMS)) [18, 26, 33]. Six items were selected as relevant for our study. The first two items regarding courtesy and respect were combined into “you were treated with less courtesy or respect than others”. The original item “you received poorer service than others” was rephrased into “you received a poorer medical treatment than others”, and three items were introduced without any modification (“people acted as if you are not smart”, “people acted as if they are afraid of you”, and “people acted as if they are better than you”). Thus, the scale in the present survey consists of five items in total (see Supplementary Material 1). The Cronbach’s alpha in our study (α = 0.72) is close to the satisfactory results of the initial work by Bird et al. [26] (α = 0.78). Introductorily, respondents were asked: “How often have any of the following things happened to you in a medical practice?”. The question was meant to include all medical professions in outpatient care. Participants without any utilization of outpatient care were excluded. Answer categories were “never”, “rarely”, “sometimes”, “often”, and “very often”. A sum scale was calculated using the situational coding approach [34]. To this end, each item was dichotomised to ‘never’= 0 and ‘ever’= 1 including those reporting “rarely” or more often. Items were then summed (range: 0–5) to assess the total number of different situations ever experienced in a medical practice.

The following social characteristics of the respondents were considered as covariates: age, sex, education, income, and migration history. Age was categorized into three age groups: 18–40 years, 41–59 years, and > 60 years. Educational level was assessed using the established CASMIN educational classification which is a hierarchically structured measurement of certificates including the general and vocational qualifications [35]. The nine original CASMIN-levels were merged into three educational groups (low/intermediate/high). To consider household size and composition, monthly net household income was equalized and further divided into tertiles. In terms of migration history, respondents were classified into three groups: people who have immigrated themselves (1st generation); people who were born in Germany, but whose parents (one or both) have immigrated (2nd generation), and those without a migration history. Furthermore, insurance status (statutory/private) was introduced. In Germany, health insurance is compulsory and characterized by a dual structure of statutory health insurance (SHI) and substitutive private health insurance (PHI). Citizens with an income over a certain limit, self-employed, and public servants can choose a private health insurance for substitutive full coverage. Around 11% of the population is covered through PHI [36].

### Analyses

First, prevalences of patient non-adherence and interpersonal discrimination in outpatient care were calculated. Second, crosstabs were calculated to examine bivariate associations between the different discrimination experiences and the three indicators of non-adherence. Third, multiple regression analyses were conducted with non-adherence issues as dependent variables and the number of discrimination experiences (sum scale) as independent variable adjusted for social characteristics and health insurance as covariates. Odds ratios (OR), 95%-confidence intervals (95%-CI), and p-values are documented. Analyses were carried out using the Statistical Package for the Social Sciences SPSS 29 [37].

## Results

Detailed information about the sample characteristics and the distribution of variables is shown in Table 1. Depending on the different experiences of discrimination, prevalences of reported discrimination vary between 4.8% (afraid of you) and 41.3% (better than you). 59.9% reported at least one type of perceived discrimination, 34.4% two or more. In terms of patient adherence, 23.6% did not follow doctor’s advice, 10% did not come for a follow-up visit, and 21.2% did not fill a prescription or obtain recommended drugs in the past 12 months.

**Table 1:** Sample description and distribution of variables (*N* = 3,246)^a^

Variables (number of missing cases in brackets/italics)		*n* (%)
Sex *(0)*	Female	1,661 (51.2)
	Male	1,585 (48.8)
Age groups *(0)*	18–40	1,078 (33.2)
	41–60	1,103 (34.0)
	> 60	1,065 (32.8)
Migration history *(0)*	No	2,451 (75.5)
	1 st generation	289 (8.9)
	2nd generation	506 (15.6)
Education *(31)*	Low	860 (26.8)
	Intermediate	1,021 (31.8)
	High	1,333 (41.4)
Income *(377)*	Low	952 (33.2)
	Intermediate	907 (31.6)
	High	1,010 (35.2)
Health insurance *(8)*	Statutory	2,870 (88.6)
	Private	368 (11.4)
Discrimination experiences (ever) *(109*^*b*^*)*	Less courtesy/respect	849 (27.1)
	Poorer treatment	696 (25.8)
	Not smart	825 (26.0)
	Afraid of you	152 (4.8)
	Better than you	1303 (41.3)
Number of different discrimination experiences (ever) *(35)*	0	1288 (40.1)
	1	820 (25.5)
	≥ 2	1103 (34.4)
Non-adherence (past 12 months) (*488-622*^c^)	Not following doctor’s advice	618 (23.6)
	Not coming for a follow-up visit	267 (10.0)
	Not filling a prescription or obtaining recommended drugs	584 (21.2)

^a^ weighted

^b^ including respondents who have never been in a medical practice

^c^ including respondents without need for medical care and without doctor advice/follow-up visit/prescribed or recommended drugs in the past 12 months

In Table 2, bivariate associations between interpersonal discrimination in outpatient care and non-adherence are shown. Four of the five discrimination experiences were significantly associated with all three adherence issues indicating higher non-adherence among patients who reported discrimination. For example, respondents who were treated with less courtesy or respect more often ignored doctor’s advice, failed a follow-up visit, and neglected prescriptions or recommended drugs than participants without this discrimination experience (*p* < 0.001). Furthermore, a trend is shown that the more discrimination experienced, the higher the likelihood of being non-adherent.

**Table 2:** Different interpersonal discrimination experiences in outpatient care and non-adherence (*N* = 2,209-2,726): n (%)

	Patient non-adherence					
	Did not follow doctor’s advice		Did not come for a follow-up visit		Did not fill a prescription or obtain recommended drugs	
Discrimination experiences		p (χ^2^)		p (χ^2^)		p (χ^2^)
*Less courtesy and respect*						
never	405 (21.5)	< 0.001	168 (8.8)	< 0.001	364 (18.5)	< 0.001
ever	203 (29.5)		96 (13.7)		203 (27.9)	
*Poorer treatment*						
never	338 (20.6)	< 0.001	149 (9.0)	0.002	297 (17.4)	< 0.001
ever	183 (32.3)		78 (13.5)		179 (29.4)	
*Not smart*						
never	364 (18.8)	< 0.001	174 (8.8)	< 0.001	326 (16.1)	< 0.001
ever	248 (37.8)		92 (13.9)		244 (35.1)	
*Afraid of you*						
never	577 (23.3)	0.064	250 (9.9)	0.078	540 (20.7)	0.010
ever	34 (30.9)		17 (15.0)		35 (30.7)	
*Better than you*						
never	291 (18.9)	< 0.001	120 (7.7)	< 0.001	278 (17.4)	< 0.001
ever	315 (30.4)		145 (13.8)		287 (25.8)	
Number of different discrimination experiences						
none	163 (15.3)	< 0.001	74 (6.9)	< 0.001	146 (13.2)	< 0.001
1	159 (24.1)		64 (9.5)		148 (21.3)	
≥ 2	295 (33.3)		129 (14.4)		286 (30.2)	

Table 3 shows the results of multiple logistic regressions. With one exception, there were highly significant associations of perceived discrimination in outpatient care with non-adherence (significant odds ratios vary between 1.77 and 2.58). Moreover, odds ratios were higher when reporting two or more discrimination experiences compared to just one. Odds ratios of the covariates show that younger age of the respondents was associated with all three adherence issues, an own migration history with medication non-adherence, and lower income with not following doctor’s advice and medication non-adherence. Moreover, lower education was associated with medication adherence while sex and health insurance status were irrelevant.

**Table 3:** Number of different discrimination experiences in outpatient care and non-adherence (*N* = 2352–2481): OR (95%-CI); *p*-values

	Did not follow doctor’s advice		Did not come for a follow-up visit		Did not fill a prescription or obtain recommended drugs	
	OR (95%-CI)	p	OR (95%-CI)	p	OR (95%-CI)	p
Different Discrimination experiences						
1	1.77 (1.37–2.29)	< 0.001	1.20 (0.82–1.77)	0.348	1.83 (1.40–2.39)	< 0.001
≥ 2	2.33 (1.84–2.95)	< 0.001	2.18 (1.57–3.03)	< 0.001	2.58 (2.02–3.30)	< 0.001
Sex						
female	1.22 (1.00-1.49)	0.055	1.00 (0.76–1.32)	1.000	1.11 (0.90–1.36)	0.337
Age						
18–40 years	1.83 (1.39–2.42)	< 0.001	2.15 (1.42–3.24)	< 0.001	2.10 (1.58–2.79)	< 0.001
41–60 years	1.26 (0.98–1.62)	0.075	2.02 (1.40–2.92)	< 0.001	1.59 (1.22–2.07)	< 0.001
Migration history						
1 st generation	1.21 (0.87–1.69)	0.266	0.97 (0.59–1.58)	0.892	1.85 (1.34–2.56)	< 0.001
2nd generation	1.09 (0.83–1.43)	0.544	0.88 (0.59–1.31)	0.528	1.16 (0.88–1.52)	0.291
Education						
intermediate	0.96 (0.76–1.23)	0.754	1.17 (0.84–1.65)	0.352	0.90 (0.71–1.68)	0.396
low	0.88 (0.66–1.18)	0.398	1.12 (0.75–1.67)	0.586	0.66 (0.49–0.89)	0.006
Income						
intermediate	1.16 (0.90–1.48)	0.251	1.27 (0.86–1.75)	0.259	1.19 (0.93–1.54)	0.168
low	1.28 (1.00-1.62)	0.049	1.40 (0.99–1.97)	0.054	1.31 (1.02–1.68)	0.032
Health insurance						
statutory	0.87 (0.63–1.19)	0.384	0.74 (0.48–1.14)	0.168	0.88 (0.63–1.22)	0.437

Reference categories: no discrimination experiences, male, >60 years, no migration history, high education, high income, private health insurance

## Discussion

In the present study, associations between five different situations of interpersonal discrimination in outpatient care and three indicators of patient non-adherence were examined in a large population sample. Results showed that reporting discrimination experiences was significantly associated with all indicators of non-adherence (not following doctor’s advice, not coming for a follow-up visit, not filling a prescription or obtaining recommended drugs). Moreover, there was a trend indicating the more discrimination experiences reported, the higher the likelihood to be non-adherent. Depending on the adherence issue, lower age, lower income, higher education, and an own migration history showed significant associations. Prevalences regarding discrimination experiences are in line with results from an US-American population survey using the EDS [25]. Only one item (better than you) revealed significantly higher percentages in the present study. Furthermore, social determinants of non-adherence in our study (younger age, lower income, and migration history) are in line with former findings [10, 11]. Previous studies from the U.S. that surveyed associations between perceived discrimination in health care and non-adherence in the general population also showed significant associations [29, 30]. Overall, research is very deficient and findings from one country cannot readily be generalized or transferred to another as discrimination as well as non-adherence vary between countries and health care systems [38, 39]. To the best of our knowledge, this is the first respective study from Germany. The results showed a clear association between perceived interpersonal discrimination and non-adherence, even when controlling for social characteristics that have previously been found to predict non-adherence [11]. Given the fact that four out of five situations of discrimination were significantly related to all three types of non-adherence (one discriminating situation was solely associated with lower medication adherence) interventions are essential. The findings of the study suggest the adverse consequences of interpersonal discrimination on illness behaviour [40], even though the direction of causality could not be identified with the cross-sectional study design. Negative emotional states caused by discrimination can lead to increased risky health behaviours, including reduced utilization of and engagement with health care services [41]. Increased unmet need and poorer doctor-patient interaction (e.g. less involvement in decision making, not having enough time with the physician) were shown to be associated with perceived interpersonal discrimination in various countries [20, 23, 25, 28, 42]. A further consequence of discrimination is non-adherence that is also associated with worse communication between physician and patient [43], and particularly, with various adverse health outcomes [9, 44]. Thus, it can be assumed that discrimination in health care can potentially affect treatment success. In terms of implications, tackling interpersonal discrimination and stereotype threat in health care should be a major goal in medical education and training [28, 40]. Interventions that reduced stereotype threat range from education about potential effects of discrimination on patients’ performance to trainings on interaction between patient and provider [40]. The individuals’ perceptions of the doctor-patient relationship is known to be a key issue in terms of non-adherence [39]. Physician communication skill trainings have been found to positively influence patient adherence which is an important predictor for health outcomes [43, 45]. Finally, the fact that medication non-adherence was more likely among less affluent respondents and people who have immigrated themselves could additionally be based on financial burden due to out-of-pocket payments.

## Limitations

There are some methodological aspects of this study that have to be discussed. First, analyses were based on an online survey. Although the sample was randomly drawn from a high-quality panel which was recruited offline, an internet connection was required to participate which potentially exclude certain groups (e.g. very old people). Second, as only about 40.4% of the invited persons participated, a selection bias cannot be ruled out. To meet potential bias, data was weighted by age, sex, federal state, and education according to the official statistics [29] using an iterative proportional fitting approach [46]. Third, analyses were restricted to individuals who were able to read German. Thus, there is a selection bias regarding respondents with a migration history, and experiences of interpersonal discrimination based on ethnicity, country of origin, or language problems can be expected to be underestimated. Fourth, although we used established scales to assess perceived discrimination, validated studies among German-speaking samples are rare [47]. Previous findings showed that associations between perceived discrimination and adherence during the past 12 months occurred independent of a specific time frame for discrimination experiences [30]. Thus, we asked if respondents ever experienced interpersonal discrimination in outpatient care. However, due to this potentially long period of time a recall bias cannot be ruled out. Finally, our cross-sectional design is not appropriate to examine causality. However, in the systematic review by Thorburn & Lindly [18], it was shown that 19 out of 22 studies examining associations between discrimination in health care and health or health care outcomes were cross-sectional. So, the study design is widespread for research questions like in our study. Nevertheless, longitudinal design and mixed-methods approaches could provide more detailed information about these associations.

## Conclusions

The present study highlights a critical issue regarding health care delivery in Germany and contradicts the goals of health equity. It shows a significant association between interpersonal discrimination experiences in outpatient care and non-adherent behaviour after controlling for social characteristics. Thus, discrimination reinforces illness behaviour that is known to be detrimental for treatment outcomes. This underlines the need to reduce discrimination due to various reasons in health care. Respective interventions include courses to reduce stereotypes in medical education and in continuing medical training.

## Supplementary Information

Supplementary Material 1.

Supplementary Material 2.

## Data Availability

Data is provided within the manuscript or supplementary information files.

## Abbreviations

SES: Socioeconomic status
EDS: Everyday Discrimination Scale
DMS: Discrimination in Medical Settings Scale (DMS)
CASMIN: Comparative Analysis of Social Mobility in Industrial Nations
SHI: Statutory health insurance
PHI: Private health insurance
OR: Odds ratio
CI: Confidence interval
