# Supplementary material for: Interpersonal discrimination experiences in outpatient care are associated with non-adherence – results of a population survey in Germany
Source: BMC Public Health. 2025 Aug 7;25:2695. doi: 10.1186/s12889-025-23951-2 (PMC12329932; doi:10.1186/s12889-025-23951-2)
Supplement: Supplementary file 1 — Supplementary Material 1. [file 12889_2025_23951_MOESM1_ESM.docx]

**SUPPLEMENTARY FILE** **1**

**S1: Items used to assesses interpersonal discrimination in outpatient care**(adapted from Peek et al. 2011)

How often have any of the following things happened to you in a medical practice?

1. You were treated with less courtesy or respect than others.
2. You received a poorer medical treatment than others.
3. People acted as if you are not smart.
4. People acted as if they are afraid of you.
5. People acted as if they are better than you.

Answer categories after each item:
never/rarely/sometimes/often/very often/not applicable/don’t know or not specified

Peek ME, Nunez-Smith M, Drum M, Lewis TT. Adapting the Everyday Discrimination Scale to medical settings: reliability and validity testing in a sample of African American patients. Ethn Dis. 2011;21:502–9.
